# Strain and Grain Size Determination of CeO_2_ and TiO_2_ Nanoparticles: Comparing Integral Breadth Methods versus Rietveld, μ-Raman, and TEM

**DOI:** 10.3390/nano11092311

**Published:** 2021-09-06

**Authors:** Yamerson Canchanya-Huaman, Angie F. Mayta-Armas, Jemina Pomalaya-Velasco, Yéssica Bendezú-Roca, Jorge Andres Guerra, Juan A. Ramos-Guivar

**Affiliations:** 1 Laboratorio de No Metálicos, Facultad de Ingeniería Química, Universidad Nacional del Centro del Perú (UNCP), Av. Mariscal Ramón Castilla No. 3909, El Tambo, Huancayo 12000, Peru; yamerson2016@gmail.com (Y.C.-H.); armasfiorella23@gmail.com (A.F.M.-A.); jpomalayavelasco@gmail.com (J.P.-V.); ybendezu@uncp.edu.pe (Y.B.-R.); 2 Departamento de Ciencias, Sección Física, Pontificia Universidad Católica del Perú, Av. Universitaria 1801, Lima 15088, Peru; guerra.jorgea@pucp.edu.pe; 3 Grupo de Investigación de Nanotecnología Aplicada para Biorremediación Ambiental, Energía, Biomedicina y Agricultura (NANOTECH), Facultad de Ciencias Físicas, Universidad Nacional Mayor de San Marcos, Av. Venezuela Cdra 34 S/N, Ciudad Universitaria, Lima 15081, Peru

**Keywords:** X-ray diffraction, CeO_2_, TiO_2_, crystallite size, strain, TEM, μ-Raman

## Abstract

Various crystallite size estimation methods were used to analyze X-ray diffractograms of spherical cerium dioxide and titanium dioxide anatase nanoparticles aiming to evaluate their reliability and limitations. The microstructural parameters were estimated from several integral breadth methods such as Scherrer, Monshi, Williamson–Hall, and their variants: (i) uniform deformation model, (ii) uniform strain deformation model, and (iii) uniform deformation energy density model. We also employed the size–strain plot and Halder–Wagner method. For this purpose, an instrumental resolution function of an Al_2_O_3_ standard was used to subtract the instrumental broadening to estimate the crystallite sizes and strain, and the linear regression analysis was used to compare all the models based on the coefficient of determination. The Rietveld whole powder pattern decomposition method was introduced for comparison purposes, being the best candidate to fit the X-ray diffraction data of metal-oxide nanoparticles. Refined microstructural parameters were obtained using the anisotropic spherical harmonic size approach and correlated with the above estimation methods and transmission electron microscopy images. In addition, μ-Raman spectra were recorded for each material, estimating the mean crystallite size for comparison by means of a phonon confinement model.

## 1. Introduction

Nanoparticles (NPs) can be obtained through various physical, chemical, or biological synthesis methods [1]. Details of the NPs manufacturing process are essential since they may affect the photocatalytic, adsorptive, thermal, and optical properties of metal oxides such as cerium oxide (CeO_2_) and titanium oxide (TiO_2_), which depend on the particle size, shape, and crystal morphology [2]. Hence, the possibility of tuning structural and morphological properties is highly relevant for the optimal use of NPs in different applications. For example, CeO_2_ NPs are widely used due to their wide range of applications in electrochemistry, such as electrode materials in supercapacitors and medicine, due to their antibacterial properties [3]. Additionally, TiO_2_ NPs are broadly applied in photocatalysis, solar cells, biomedicine, chemical sensors, lithium storage [2] and have also been explored for heavy metal water cleaning purposes [4].

Currently, physical techniques such as scanning electron microscopy (SEM), transmission electron microscopy (TEM), and atomic force microscopy (AFM) are used to estimate and confirm the NPs’ scale [5], as well as indirect methods such as powder X-ray diffraction (PXRD). In particular, PXRD analysis is widely used for determining crystallite sizes and lattice deformation. The information of the two latter physical parameters correlates with the diffraction peak broadening with lattice strain arising from imperfections (stacking faults or coherency stresses) of the studied material. There exist several methods to estimate the peak broadening from XRD data [6]. However, the accuracy of these methods is questionable due to inconsistencies between them [6].

The above-mentioned PXRD methods used nowadays are modifications of the Scherrer method (1918), which relates the peak broadening (*β*) of each Bragg peak to a characteristic mean size (*D*) [7]. Since that first work, new parameters have been added to better represent the physical effects that can be produced in the intensity distribution of diffracted X-rays. In 1953, the Williamson–Hall (W–H) method introduced the general distribution of deformations *ε* [8], which takes into account the two-parameter effects of size and lattice strain. Generalized models, such as the uniform deformation model (UDM), considers the uniform stress in all crystallographic directions, considering an isotropic crystal [2]. Whilst the mechanical Hooke’s law considers the strain in the uniform deformation stress model (USDM) and uniform deformation energy density model (UDEDM), the latter methods take into account the anisotropic nature of Young’s modulus of the crystal [9,10]. Instead, the Halder–Wagner (H–W) method assumes that the spreading of the peak is a symmetric Voigt function, which means that the crystallite size is defined by the Lorentzian and Gaussian functions [11]. Similarly, the size–strain plot (SSP) method considers that the X-ray profile is described by a linear combination of Lorentz and Gaussian functions [12].

On the other hand, the Rietveld method (RM) is a tool for analyzing crystalline structures [13], which is based on the theoretical refinement of the structural or cell parameters, atomic displacements, anisotropy, cell stresses, shape, and anisotropy effects, among others, leading to a convergence between the values of the experimental diffractogram curves and the theoretical model [14]. Further, this refinement is suitable when making a multiphasic analysis (percentual phase concentration). For example [15], when diffraction peaks overlap, systematic errors can arise from the incorrect determination of full width at half maximum (FWHM) values, as can be the case in previously discussed methods. The theoretical model includes structural aspects such as crystalline structure, spatial group, atom Wyckoff positions, etc. In addition, microstructural information is involved, including crystal size and micro-deformations. The RM also includes instrumental factors such as the optical effect of XRD equipment on the width of diffraction peaks [16]. It is worth mentioning that the RM makes use of known atomic structures to generate an initial theoretical model of the structure of the phases present in the sample, so these must be previously identified. From this initial model, the method allows refining the structural parameters based on the analysis of least squares [17] until the model matches the experimental profile [16].

In the present work, a comparative study of the microstructural parameters of metal-oxide CeO_2_ and TiO_2_ NPs has been investigated by employing different modified models, including W–H, UDM, UDSM, UDEDM, SSP, and H–W. All the methods were compared to the obtained results of RM (spherical harmonic approach) and TEM images. Additionally, crystallite sizes were also estimated from μ-Raman measurements by means of a phonon confinement model.

## 2. Materials and Methods

### 2.1. X-ray and TEM Experimental Details

TiO_2_, CeO_2_, and standard aluminum dioxide (Al_2_O_3_) were obtained from Sigma Aldrich (Burlington, MA, USA). No further purification was performed of the powder samples. The PXRD data were collected using a Rigaku diffractometer (Tokyo, Japan), operating with CuKα radiation (1.5406 Å) at 50 kV and 100 mA. The diffractograms were collected using a step scanning configuration between *2θ* = 20°–100° for CeO_2_ and TiO_2_, with 0.02° and 5s per step. The crystallographic phases were identified using Match-Phase Identification from Powder Diffraction software (version3, Crystal Impact, Germany) using the crystallographic cards (96-434-3162) and (96-500-022) with the crystallographic information files (CIF) #9009008 and #5000223 for the CeO_2_ and TiO_2_ anatase phases, respectively. The OriginPro 9.0 software was used to estimate the FWHM, using a pseudo-Voigt fitting model corrected by the instrumental resolution function (IRF) obtained from the standard corundum (see Figure S1). 

For the RM of the diffractograms, the software FullProf Suite (version July 2001) was employed, the CeO_2_ and TiO_2_ crystallographic information files (CIF) obtained from Match v3 software were used as initial parameters, which crystallographic data for CeO_2_ were cubic crystalline structure, space group Fm-3m, and cell parameter a = 5.4110 Å. For TiO_2_ anatase, they were tetragonal crystalline structure, space group I 41/amd, cell parameters a = 3.78435 Å and c = 9.50374 Å. For both cases the Caglioti initial parameters were U = 0.004133, V = −0.007618, and W = 0.006255. Refinement was done using the Thompson–Cox–Hastings (TCH) pseudo-Voigt Axial divergence asymmetry function. Finally, the average crystallite size was determined in the FullProf Suite program. To do that, we first characterized the Al_2_O_3_ standard. The used experimental conditions were *2θ* = 10°–80° with a step of 0.02°. For the Al_2_O_3_ refinement, the TCH profile was employed to obtain the instrumental parameters of the equipment, which was added to the instrumental resolution file (IRF) and later used to determine the average crystallite sizes of the CeO_2_ and TiO_2_ NPs.

### 2.2. μ-Raman Experimental Details

Structural and vibrational features of the nanopowders were analyzed by Raman spectroscopy using a confocal µ-Raman microscope inVia^TM^ by Renishaw (Edinburgh, UK). The spectrometer was configurated with a 1200 grooves/mm diffraction grating and a ×50 objective with N.A. 0.75 and a working distance of 0.37 mm. The excitation wavelength was set to 785 nm from a laser diode. The laser power was set to ~1 mW. After identifying the main Raman optical modes of vibration, we used a phonon confinement model (PCM) for the estimation of nanocrystals size [18,19].

## 3. Results and Discussion

### 3.1. PXRD Analysis

PXRD diffractograms for CeO_2_ and TiO_2_ NPs are shown in Figure S2a,b. For CeO_2_, it indicated a monophase that could be indexed to a cubic structure. Figure S2b shows the TiO_2_ diffractogram, which only detected the Bragg peaks of the anatase phase [20] as there were no diffraction lines that have a rutile-phase TiO_2_.

### 3.2. Scherrer Method

**Figure 1:**
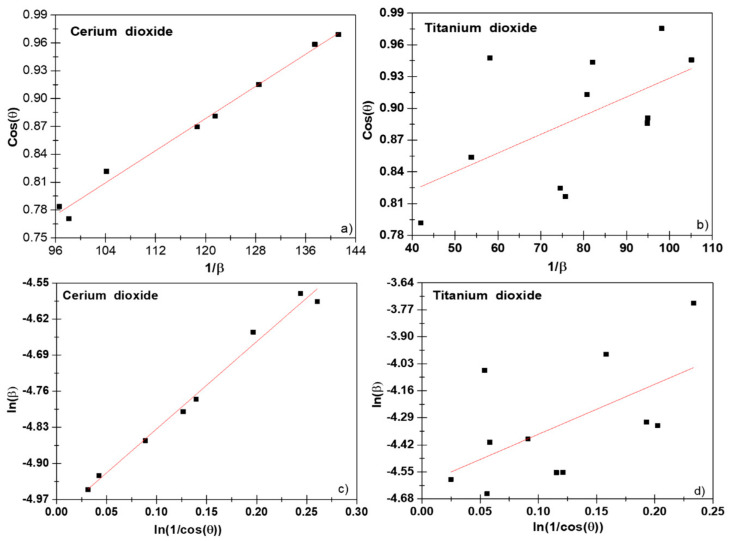
Scherrer plot (**a**,**b**) and modified Scherrer plot (**c**,**d**) of CeO_2_ and TiO_2_ NPs.

Scherrer obtained his equation for ideal conditions like parallel, infinitely narrow, and monochromatic X-ray beam diffracting on a monodisperse powder of defined crystallites with uniform coherent domains [21]. The broadening of the diffraction peak in the nanocrystals is exclusively related to the crystallite size, and non-intrinsic strain effects are considered. This broadening often consists of physical and instrumental broadening parts; the latter can be corrected with the following relationship [22]:

(1) $$\;\beta_{D}={\left[\beta_{measured}^{2}-\beta_{instrumental}^{2}\right]}^{1/2}\;\;$$

where $\;\beta_{D}$ is the corrected peak broadening. The instrumental broadening and physical broadening of the sample were measured as FWHM. So, with the Scherrer method, we could calculate the average particle size and ignoring the contribution of the strain, the average crystallite size was calculated by the following equation:

(2) $$\;D=\frac{K\lambda }{\beta_{D}cos\theta }\;$$

where K is the morphological parameter or shape factor for spherical particles equal to 0.94 nm^−1^, the wavelength (*λ*) of the radiograph is 1.54056 Å for ${\mathrm{CuK}}_{\alpha 1}$ radiation, the Bragg diffraction angle (*θ*) and the FWHM is rewritten as $\beta_{D}$ and expressed in radians. The plot $1/\beta_{D}$ vs. cosθ shown in Figure 1a gave R^2^ values higher for the CeO_2_ NPs than TiO_2_ NPs (Figure 1b). To obtain accurate results, it is important to highlight that the Scherrer equation could only be used in the next cases: (i) for average sizes up to 100–200 nm; (ii) sample and signal/noise ratio, because the broadening of the XRD peak decreased as the crystallite size increased and it was difficult to separate the broadening from the peak [21]; and (iii) since a line profile standard could not have residual broadening due to the domain size or strain, or other sources, a well-crystallized powder had to be employed.

### 3.3. Monshi Method

Monshi [7] introduced some slight modifications to Equation (2). As reported by Rabiei et al. [23], the use of Scherrer’s equation gave an increment in the estimation of the nanocrystalline sizes when the *2θ* values increased (typical positive linear curve). Then, the introduction of $\sum {\left(\pm ∆ln\beta \right)}^{2}\;$ yielded more reasonable values of *D*, with the next modified equations [23,24]:

(3) $$\;\beta_{D}=\frac{K\lambda }{Dcos\theta }=\;\frac{K\lambda }{D}\frac{1}{cos\theta }\;\;$$

(4) $$ln\beta_{D}=ln\left(\frac{K\lambda }{D}\right)+ln\left(\frac{1}{cos\theta }\right)\;$$

From the plot of $ln\left(\frac{1}{cos\theta }\right)$ vs. $ln\beta_{D}$, we could observe a straight line with an intercept of $ln\left(\frac{K\lambda }{D}\right)$, from which an average *D* value could be calculated, see Figure 1c,d. As we can see in Figure 1d, the value of R^2^ increased compared to the results from the Scherrer equation. This is because there were various peaks in the range of *2ϴ* = 20°–80°, and it was assumed that all these peaks should represent equal values for the crystallite size domains. This correction gave us an increment in the *D* values, obtaining 21.6 (3) nm for CeO_2_ and 14.6 (2) nm for TiO_2_ NPs when comparing them with the Scherrer method that yielded values of 19.6 (2) nm for CeO_2_ and 12.7 (2) nm for TiO_2_ NPs, respectively.

### 3.4. W–H Method 

In comparison to the Scherrer method, the W–H method considers the effect of strain on the XRD peak broadening and, therefore, can be used for the estimation of the intrinsic strain separated from the *D* value. Then, the total broadening can be written as [22]:

(5) $$\;\;\beta_{total}=\beta_{size}+\beta_{strain}\;$$

where $\beta_{size}$ is the broadening due to size and $\beta_{strain}$ is related to the strain broadening contribution. In the next section, we analyze the crystallite size and micro-deformation using the modified W–H equation as UDM, USDM, and UDEDM.

#### 3.4.1. UDM Method

UDM allowed for a uniform deformation along the chosen crystallographic direction, which was assumed to be isotropic. This intrinsic deformation played an important role in the broadening of the XRD profile, which was defined as the broadening *β_s_* that was related to the effective stress and Bragg angle by the equation [25]:

(6) $$\beta_{strain}=4\varepsilon tan\theta \;$$

where the deformation *ε* can be calculated from the expression $\varepsilon =\frac{\beta_{hkl}}{4tan\theta }$. Therefore, the total broadening $\;\beta_{hkl}$ representing the FWHM of a diffraction peak due to the contribution of the lattice strain $\beta_{strain}$ and the size of the $\beta_{size}$ crystallites in a particular peak that can be expressed as:

(7) $$\;\beta_{hkl}=\beta_{strain}+\beta_{size}$$

(8) $$\;\;\;\beta_{hkl}=\frac{K\lambda }{Dcos\theta }+4\varepsilon tan\theta \;\;\;$$

Equation (8) can be mathematically represented by:

(9) $$\;\;\beta_{hkl}cos\theta =\frac{K\lambda }{D}+4\varepsilon sin\theta \;\;$$

**Figure 2:**
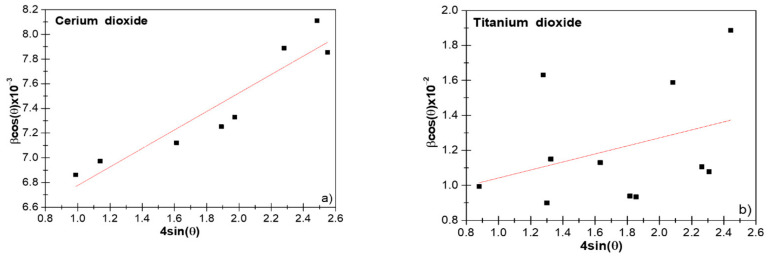
The Williamson–Hall (W–H) analysis of CeO_2_ (**a**) and TiO_2_ (**b**) nanoparticles (NPs), assuming uniform deformation model UDM.

From the slope of the straight line between *4sinθ* and $\beta_{hkl}$*cosθ*, the strain (*ε*) could be estimated, and the average crystallite size could be estimated by the extrapolation of the Y-intercept Equation (10); see Figure 2a,b.

(10) $$\;D=\frac{K\lambda }{intercept\;\left(y\right)}\;$$

By using the IRF, we obtained the average crystallite size of the y-intercept from the linear fit. Values of 24 (9) nm for the CeO_2_ and 17.9 (8) nm for TiO_2_ NPs were obtained, respectively. In principle, this method was not realistic at all due to its supposed isotropic nature. Notice that crystalline domains were assumed to be spherical and, hence, were independent of (hkl) [26].

#### 3.4.2. USDM Method

As we recall from Hooke’s law, in the framework of the elastic limit, there exists a linear relation between *ε* and stress (*σ*), expressed by the next mathematical relation $\sigma =y_{hkl}\varepsilon$, where $y_{hkl}$ represented Young’s modulus. By replacing *ε = σ/*$y_{hkl}$ in Equation (9), we have the next relation:

(11) $$\;\beta_{hkl}cos\theta =\frac{K\lambda }{\beta_{D}}+\frac{4\sigma sen\theta }{y_{hkl}}\;$$

By looking into Equation (11), we noticed that $y_{hkl}$ depends on the crystallographic direction perpendicular to the set of planes (hkl). Hence, the expressions for $y_{hkl}$ in the cubic and tetragonal crystal systems must be obtained. A theoretical model for the determination of elastic constants of cubic crystals was proposed by Jamal et al. [27]. Moreover, they are related to the elastic compliance’s constants ($s_{ij}$) and stiffness constants ($c_{ij}$), as we saw below.

In case of a cubic crystal, $y_{hkl}$ is calculated using the following equation [27]:

(12) $$\frac{1}{y_{hkl}}=S_{11}-2\left[(S_{11}-S_{12})-\frac{1}{2}S_{44}\right]\left[\frac{h^{2}k^{2}+k^{2}l^{2}+l^{2}h^{2}}{{\left(h^{2}+k^{2}+l^{2}\right)}^{2}}\right]$$

(13) $$\;\;S_{11}=\frac{C_{11}+C_{12}}{\left(C_{11}-C_{12}).(C_{11}+2C_{12}\right)}\;$$

(14) $$\;S_{12}=\frac{-C_{12}}{\left(C_{11}-C_{12}).(C_{11}+2C_{12}\right)}\;$$

(15) $$\;\;S_{44}=\frac{1}{C_{44}}\;$$

where the values of the elasticity stiffness constants $C_{11}$, $C_{12}$, and$\;C_{44}$ for cubic CeO_2_ were 455, 188.7, and 81.48 GPa, respectively [28]. By using these values, the elastic compliances constants had the values of $2.904\times 10^{-12}$, −8.513 $\times 10^{-13}$, and 1.227 $\times 10^{-11}$, respectively. In consequence, the estimated value of $y_{hkl}\;$for each diffraction peak was taken as the average value of 270.3 GPa. Considering a tetragonal crystal, the $y_{hkl}$ had the next mathematical relation [2]:

(16) $$\;\frac{1}{y_{hkl}}=\frac{S_{11}\left(h^{4}+k^{4}\right)+\left(2S_{12}+S_{66}\right)h^{2}k^{2}+\left(2S_{13}+S_{44}\right)\left(h^{2}+k^{2}\right)l^{2}+S_{33}l^{4}}{{\left(h^{2}+k^{2}+l^{2}\right)}^{2}}$$

where $S_{11}$, $S_{12}$, $S_{13}$, $S_{33}$, $S_{44}$, and $S_{66}$ are the elastic *ε* for TiO_2_ anatase. Their values were $5.1\times 10^{-12},-0.8\times 10^{-12},-3.3\times 10^{-12},\;10.7\times 10^{-12},\;18.5\times 10^{-12},\;\mathrm{and}\;16.7\times 10^{-12}N/m^{2}$, respectively [2,29]. Using these elastic *ε*, the value of $y_{hkl}$ for each diffraction peak was also calculated as the average value of 127 GPa.

Figure S3a,b shows a plot between $4sen\theta /y_{hkl}\;$vs. $\beta_{hkl}cos\theta ,$ a linear fit was done where the slope represents the *ε*, and the average crystallite size was calculated of the intersection with the axis obtaining a value of 22.8 (1) nm for CeO_2_ and 17 (6) nm for TiO_2_. As mentioned, in contrast to the above method, this model used the corresponding average $y_{hkl}$.

#### 3.4.3. UDEDM Method

While UDM assumes a homogeneous isotropic crystal, this homogeneity and isotropy is no longer justified for a real crystallographic system. For an anisotropic crystal, the W–H equation must be modified by anisotropic terms [8]. This was done in USDM, which assumes a linear relation between ε and σ, according to Hooke’s law. However, in real crystals, the isotropic nature and linear proportionality cannot be considered due to crystal defects, such as dislocations and agglomerations, that create imperfections in almost all crystals.

Thus, we have UDEDM, which considers the deformation of crystals, the uniform anisotropic deformation of the lattice in all crystallographic directions, and the cause of that uniform anisotropic deformation of the lattice as the deformation energy density (u). Therefore, the proportionality constants associated with the *σ*-strain relationship are left to be independent. The *ε* energy (energy per unit volume) as a function of *ε* is given by Hooke’s law as:

(17) $$\;\;u=\frac{\left(\varepsilon^{2}y_{hkl}\right)}{2}=\frac{\sigma^{2}}{2y_{hkl}}\;$$

where *σ* and *ε* are related as $\sigma =\varepsilon \times y_{hkl},\;$so the intrinsic *ε* can be written as a function of energy density.

(18) $$\varepsilon =\sigma \sqrt{\frac{2u}{y_{hkl}}}\;$$

The W–H equation is modified in UDEDM by:

(19) $$\;\;\beta_{hkl}cos\theta =\frac{K\lambda }{D}+4sen\theta {\left(\frac{2u}{y_{hkl}}\right)}^{1/2}\;$$

The plot of Equation (23), with the term $4sen\theta {\left(\frac{2}{y_{hkl}}\right)}^{1/2}$ along X-axis and $\beta_{hkl}cos\theta$ along the Y-axis corresponding to each diffraction peak, where the density of energy was obtained from the slope and the average crystallite size was taken from the y-intercept of the linear fitting. Figure S3c,d represents the UDEDM fitted plots for CeO_2_ and TiO_2_ NPs, respectively. The points were from experimental data, and the fitted data are shown as a straight line. The calculated parameters of this model are summarized in Table 1. With this method, the estimated crystallite size was 23.6 (1) and 18.5 (8) nm, with R^2^ values of 0.75 and 0.08 for CeO_2_ and TiO_2_, respectively. In comparison, this fit was not as good as those obtained with the previous methods. The *ε* values obtained for these samples were 1.26 × 10^−7^ and 7.42 × 10^−7^, and the stress values were 1.85 × 10^−4^ and 3.1 × 10^−4^ TPa for CeO_2_ and TiO_2_, respectively.

### 3.5. SSP Method 

**Figure 3:**
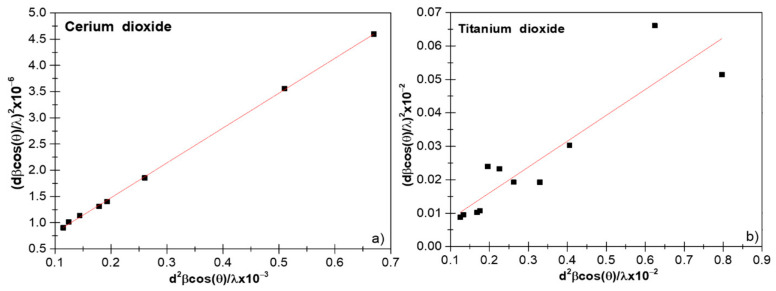
The size–strain plot (SSP) of CeO_2_ (**a**) and TiO_2_ NPs (**b**).

This method had a better result for isotropic broadening; since at higher diffraction angles, the XRD data were of lower resolution and the peaks overlapped [23]. Figure 3a,b show the linear-regression plots obtained for CeO_2_ and TiO_2_ NPs, respectively.

The SSP is one of the methods that consider the XRD peak profile to be a combination of the Lorentzian and the Gaussian functions. Under this assumption, the *ε* profile is reflected by the Gaussian function and the size of the crystallites by the Lorentz function.

(20) $$\;\beta_{hkl}=\beta_{L}+\beta_{G}$$

where $\beta_{L}$ and $\beta_{G}$ are broadening peaks due to the Lorentz and Gauss functions, respectively. The SSP equation is presented below:

(21) $$\;{\left(d_{hkl}\beta_{hkl}cos\theta \right)}^{2}=\frac{K\lambda }{D}\left(d_{hkl}{}^{2}\beta_{hkl}cos\theta \right)+\frac{\varepsilon^{2}}{4}\;\;$$

In this particular method, less weight is given to higher angle diffraction data, for which the precision is usually lower [11] since XRD peaks are often highly overlapping [22]. It was clearly observed that the average crystallite size obtained for both CeO_2_ and TiO_2_ NPs was smaller compared to Scherrer’s method; this difference can result from structural deformations. Therefore, when using a method that does not consider stress, it can give us inaccurate results [30].

### 3.6. H–W Method

**Figure 4:**
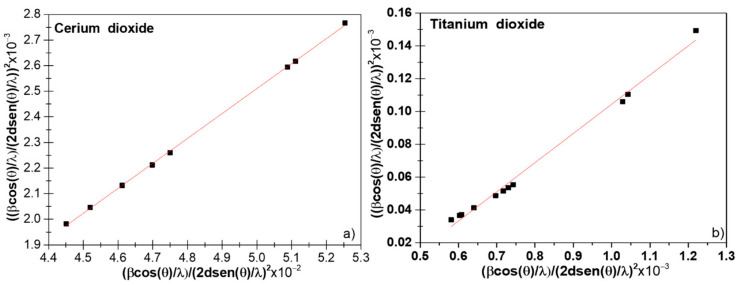
Halder–Wagner (H–W) plot of CeO_2_ (**a**) and TiO_2_ (**b**).

In the above method, the XRD peak profile size extension was assumed as a Lorentzian function, while *ε* broadening as a Gaussian function. That is why the H–W method was proposed, which is based on the assumption that peak broadening is a symmetric Voigt function, which is a convolution of the Lorentz and Gauss functions. Hence, for a Voigt function, the full width at half maximum of the physical profile can be written by H–W method as:

(22) $$\;\beta^{2}{}_{hkl}=\beta_{L}\beta_{hkl}+\beta_{G}^{2}\;$$

where $\beta_{L}$ and $\beta_{G}$ are the full widths at half maximum of the Lorentzian and Gaussian functions. This method gives more weight to Bragg peaks in the low and middle angle range, where the overlap of diffraction peaks was low, and the relationship between the size of the crystallite and the lattice *ε* according to the H–W method is given by [22]:

(23) $$\;{\left(\frac{\beta^{*}{}_{hkl}}{d^{*}{}_{hkl}}\right)}^{2}=\frac{1}{D}\times \frac{\beta^{*}{}_{hkl}}{d^{*}{{}_{hkl}}^{2}}+{\left(\frac{\varepsilon }{2}\right)}^{2}\;$$

where $\beta^{*}{}_{hkl}=\beta_{hkl}\times \frac{cos\theta }{\lambda }$ and $d^{*}{}_{hkl}=2\times d_{hkl}\times \frac{sin\theta }{\lambda }$; the plot $\frac{\beta^{*}{}_{hkl}}{d^{*}{{}_{hkl}}^{2}}$ vs. ${\left(\frac{\beta^{*}{}_{hkl}}{d^{*}{}_{hkl}}\right)}^{2}$ is shown in the Figure 4 [23]. The slope of the plotted straight line provided average crystallite sizes. From the data shown in Figure 4a,b, we obtained values of 10.3 (8) and 5.6 (2) nm for CeO_2_ and TiO_2_ NPs, respectively. From the intercept, we had the intrinsic *ε* of the nanocrystals. We can observe from Figure 4a,b that the H–W method has a better-defined linear trend than previous methods as a result of the integral broadening to a Voigt function.

By comparing the results of the crystallite size and lattice *ε*, shown in Table 1, we could see that the H–W method showed a decrease in the crystallite size. A common feature between the W–H and H–W methods was that the dispersion of data points increased with increased lattice *ε*, which would indicate that lattice *ε* was anisotropic [31]. However, in our case, we saw a decrease in the dispersion of the points and also an increase in ε, suggesting that lattice *ε* was isotropic in nature.

The R^2^ values are important to differentiate among all of the studied linear methods (see Table 1). We obtained only positive values of R^2^ for all of the crystallographic phases; the method was more accurate if the R^2^ was near 1 or, in other words, data points of x and y were closer to the fitting line [32]. Therefore, we suggest in principle that the H–W method gives the most accurate results.

### 3.7. Rietveld Refinement and Spherical Harmonic Approach

As can be observed, all the crystallite estimation methods interpreted the integral broadening considering homogeneous isotropic size in all crystallographic directions. Moreover, despite the IRF was used to obtain the physical broadening, other experimental parameters and function profile attributes were not taken into consideration when using the one- or two-parameter approaches presented in previous sections. This was reflected in the high-dispersed data and in the low values obtained for R^2^ in the case of TiO_2_ NPs, with considerable dislocations and stacking faults effects. Scardi et al. studied the modified version of the W–H method, including quadratic variation [9]. Four variant models were studied in nanocrystalline CeO_2_ [9]. Hence, they could be used only for a quick determination of ‘size’ and ‘strain’. On the other hand, the whole powder pattern modeling (WPPM) was proposed as an optimum method, including optical components and instrumental parameters in the RM. More emphasis was put on the crystallite size domain determination [26,33]. Recently, it was shown that the convolutive approach could be used to model the diffraction line profile [34] of nanomaterials. Moreover, the use of the expensive and not-free-of-charge software TOPAS was needed for the modeling of the diffraction lines with advanced included macros that accounted for the fundamental parameter approach. However, the software FullProf Suite could be used for full WPPM, where the pseudo-Voigt function was defined by a linear combination of Lorentzian and Gaussian contributions (see FullProf Suite manual [35]). Additionally, the software included the formulation by Popa that considered the anisotropic size by employing the spherical harmonic approach (SHP), as given by the formula [36]:

(24) $$\beta_{h}=\frac{\lambda }{D_{h}\cos\theta }=\frac{\lambda }{\cos\theta }\underset{\mathrm{lmp}}{\sum }a_{\mathrm{lmp}}y_{\mathrm{lmp}}\left(\Theta_{h},\Phi_{h}\right)$$

where h is a vector that represents the (hkl) indices in the reciprocal space, $\beta_{h}\;$is the size contribution to the integral breadth of reflection (hkl), y_lmp_($\Theta_{h},\Phi_{h}$) are the real components of spherical harmonics ($\Theta_{h}$ and $\Phi_{h}$ correspond to the polar and azimuthal angles of the vector in the Cartesian crystallographic frame), and a_lmp_ are the corresponding refined coefficients given in Table 2, related to the Laue class. For CeO_2_, an m$\bar{3}$m Laue Class, while for TiO_2_ anatase, a 4/mmm Laue class was considered. The values for the a_lmp_ coefficients are available in Table 2.

**Figure 5:**
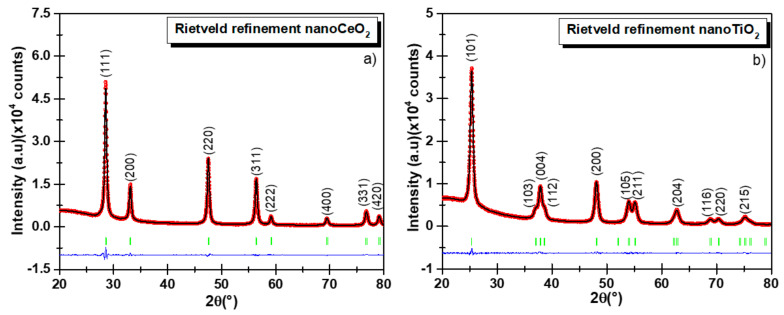
Rietveld refinement of XRD diffractograms using the Thompson–Cox–Hastings (TCH) function for CeO_2_ (**a**) and TiO_2_ NPs (**b**). The observed experimental diffractograms are given by the red lines (I_obs_), the black lines (I_cal_) are calculated diffractograms, and the residual lines are shown in blue color. The refinement parameters are reported in Table 2.

Figure 5a,b shows the refined diffractogram of CeO_2_ and TiO_2_ NPs using the TCH profile functions, and the refined parameters are displayed in Table 2. We could notice that for the low-angle region, the background was considerable and could be explained by the instrumental conditions. For the presented experimental conditions, the ‘peak data’ collection depended on the type of material, quantity, and nature of its properties, such as particle size, composition, sample preparation method, etc. Anisotropic size broadening observed in nanosamples often shows this kind of XRD diffractograms with significant broadening and complex shapes. Some examples can be found in [34,37]. Jensel et al. also obtained a similar diffractogram for 5 nm TiO_2_ anatase NPs [38]. While Mi et al. also observed an anisotropic size behavior for 22 nm Rutile TiO_2_ NPs [39]. Additionally, Scardi et al. also obtained similar diffractograms for Palladium nanocubes [34]. Further, Wang et al. [40] proposed an ellipsoid model to describe this kind of diffractograms for nickel hydroxide and beta-TiO_2_. In our case, we did not remove the background because important physical information might be lost. To model the background, we employed the WinPLOTR option available in FullProf. This option allowed us to select the desired background points and stored them in a bgr file. We used more than 60 points to model the background in both diffractograms. For CeO_2_ NPs, the RM confirmed the cubic structure of the CeO_2_ NPs where the characteristic diffraction peaks matched wells with the fluorite-structured CeO_2_ crystal, and no other peak belonging to another crystallographic phase was detected, confirming the high chemical purity of the sample. For TiO_2_ NPs, the corresponding tetragonal structure showed only the presence of the anatase phase. The statistically weighted profile residual (*R_wp_*) and the profile residual factor (*Rp*) were taken into consideration to monitor the refinement progress and also as indicators of the refinement enhancement. The goodness of refinement, χ^2^ (chi-squared), indicated the statistical error. In both samples, values of 1.69 and 2.99 were obtained using the TCH diffraction profiles for TiO_2_ and CeO_2_ NPs, respectively. For the estimation of the mean crystallite size the TCH profile was used for which an IRF file was yielded by fitting the XRD diffractogram of the Al_2_O_3_ standard, which allowed to subtract the instrumental broadening. The globally apparent sizes were 10.1 (2) and 15.4 (1) nm for the TiO_2_ and CeO_2_ nanocrystallites, respectively. These values were obtained from the domain size distribution, as reported in Table 2, which differed from the PSD histogram obtained from TEM. Here, the FullProf Suite software generated a mic file in which the anisotropic size values were summarized. 

### 3.8. μ-Raman Analysis 

For spherical particles with a diameter *D* and no vibration mode degeneration, the Raman intensity can be written as shown in Equation (25):

(25) $$I\left(\omega \right)\propto \int_{BZ}^{}d^{3}q\;\frac{{\left|C\left(q,D\right)\right|}^{2}}{{\left(\omega -\omega \left(q\right)\right)}^{2}+{\left(\frac{\Gamma }{2}\right)}^{2}}$$

Here, q is the phonon wave vector ranging in the BZ from −π/a to π/a, with *a* being the lattice parameter. The volume differential $d^{3}q$ under the spherical symmetry approximation is written as $d^{3}q=4\pi q^{2}dq$. Γ is the intrinsic mode line width of the Lorentzian response centered at $\omega \left(q\right)$, with $\omega \left(q\right)$ being the phonon dispersion relation, and $C\left(q,D\right)$ are the Fourier coefficients of a weighting function describing the confinement, i.e., enforcing the decay of the phonon wave function to a minimal value close to the nanocrystal boundary.

(26) $${\left|C\left(q,D\right)\right|}^{2}=\left(-\frac{q^{2}D^{2}}{8\beta }\right)\;$$

For particle diameters greater than *D* ≥ 10 nm [18,19,41], a Gaussian confinement weighting function is a good approximation, and therefore, the Fourier coefficients are written as given in Equation (26). The confinement factor β can range from 1 to 2$\pi^{2}$ for the Richter confinement model [42] and Campbell model, respectively [43]. In our case, we fixed β=1. Previous reports suggested that the dispersion relation for anatase TiO_2_ NPs near the E_g_ band can be modeled as $\omega \left(q\right)=\omega_{0}+\Delta (1-cos(qa))\;\mathrm{with}\;\Delta =20\;{\mathrm{cm}}^{-1}$. For the case of CeO_2_ NPs, we simply approximated the dispersion relation with a polynomial function of order 3, $\omega \left(q\right)=\omega_{0}+A_{1}q+A_{2}q^{2}+A_{3}q^{3}$. Distinct reports put $\omega_{0}$ for the F_2g_ mode between 464 and 466 cm^−1^. Here the best-fitted value was obtained for $\omega_{0}$ = 464.4 cm^−1^, in agreement with Spainer et al. [41]. However, our analysis did not include strain effects. 

To fit Equation (25), we proceeded by normalizing the Raman intensity to the peak area. We performed that with the experimental data and with Equation (25). In this work, the fitting procedure was formulated on the minimization of the unbiased squared error considering the free parameters $\omega_{0}$, L, and Γ.

**Figure 6:**
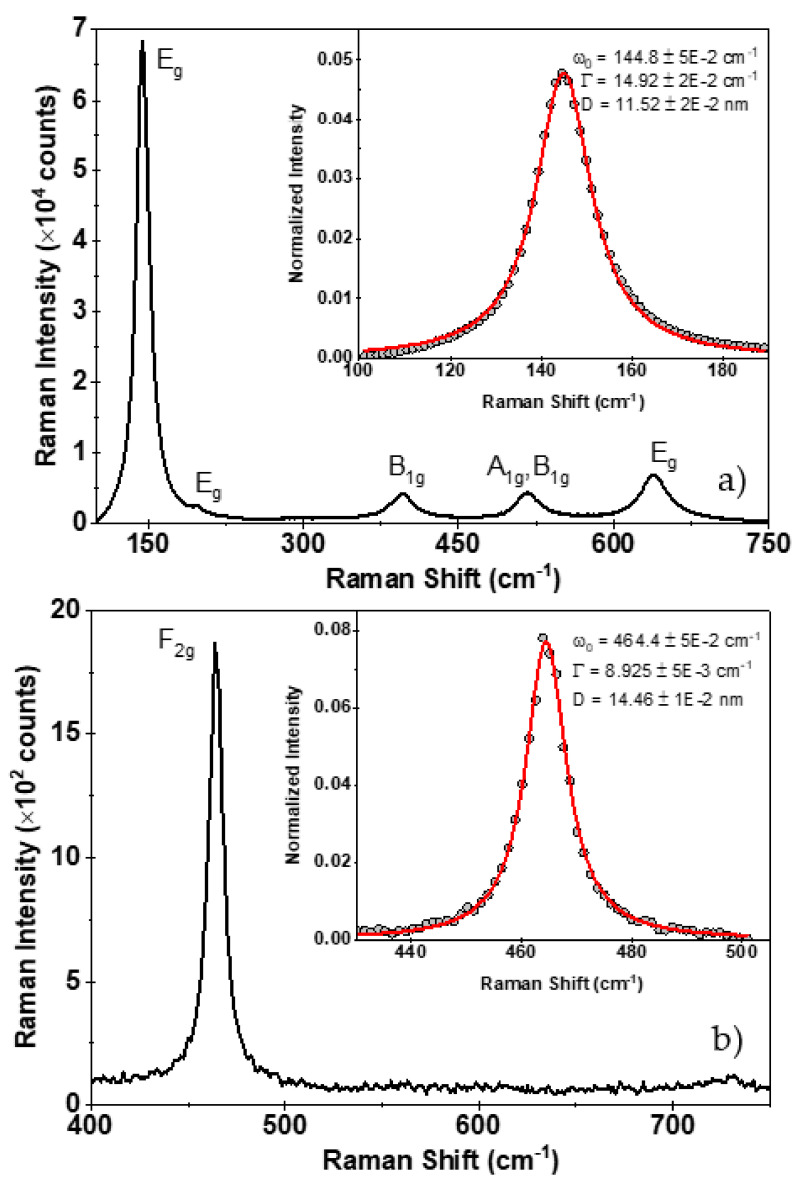
Raman spectrum of TiO_2_ (**a**) and CeO_2_ (**b**) nanopowders, respectively. Inset graphs denote the fit after Equation (25). Best fitted parameters are shown.

Figure 6a,b depict the Raman spectra of TiO_2_ and CeO_2_ NPs, respectively. For the TiO_2_ NPs, the E_g_ and B_1g_ bands were identified. The A_1g_ band could not be resolved. The analysis was performed with the E_g_ band centered at 144 cm^−1^. The characteristic F_2g_ band at 464 cm^−1^ of the CeO_2_ particles was observed and used for the analysis. Both peaks were fitted after normalization using Equation (26). The estimated NPs size were 11.5 nm and 14.5 nm for TiO_2_ and CeO_2_, respectively, with a fitting error close to 0.01 nm. An additional error source could be attributed to a particle size distribution not considered in this analysis.

### 3.9. TEM Analysis and Comparison

**Figure 7:**
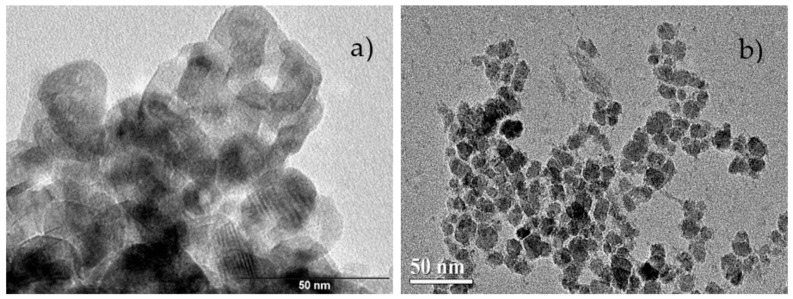
Transmission electron microscopy (TEM) image of nanoTiO_2_ (bar length of 50 nm; **a**) and nanoCeO_2_ (bar length of 50 nm; **b**).

**Figure 8:**
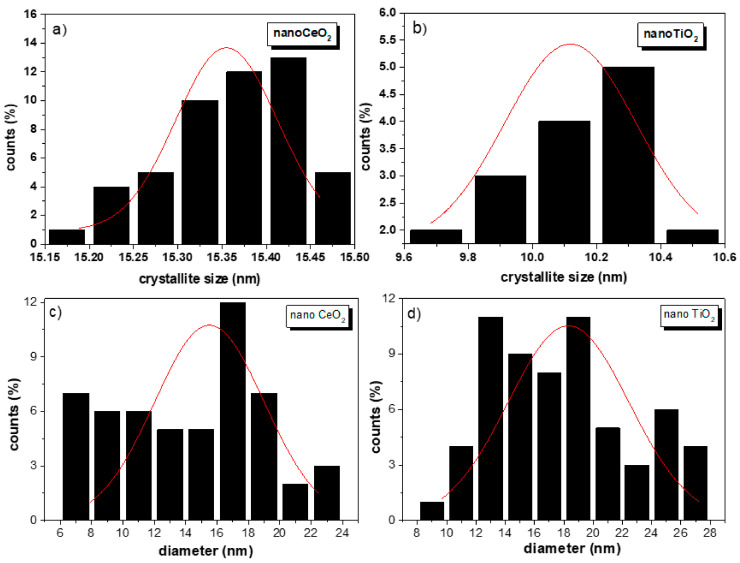
Domain size distribution and particle size distribution (PSD) histogram for nanoCeO_2_ (**a**,**c**) and nanoTiO_2_ (**b**,**d**).

TEM images for nanoTiO_2_ and nanoCeO_2_ are shown in Figure 7a,b. In the case of the nanoCeO_2_, the crystallite estimation methods gave values higher than 15 nm, overestimating the TEM value. It is important to highlight that the SHP model (15.4 (1) nm, see Figure 8a) was close to the estimated Raman value that was equal to the TEM value, suggesting the improvement in the presented model to get accurate values for CeO_2_ NPs. On the other hand, quasi-spherical TiO_2_ NPs showed a particle size of 17.9 (5) nm (Figure 8d) with mean pore sizes of 5–7 nm and a polydispersity index (PDI) value of 0.3. By comparing this with the size estimation methods, the crystallite domain size distribution gave a value of 10.1 (2) nm (Figure 8b). In contrast, the UDM method gave the same value estimated by TEM. It is clear from the particle size distribution (PSD) histogram that both NPs had a broad particle size distribution (PDI > 0.3) [44]. Nevertheless, CeO_2_ NPs presented a well-defined morphology and more homogeneous distribution in comparison to TiO_2_ NPs. Therefore, it can be concluded that, in the case of PSD, the NPs were formed by nanocrystallites with an anisotropic size behavior, and a crystallite size distribution was expected. By comparing all the methods, it can be said that the crystallite sizes obtained by the W–H method were more accurate, but an overestimation of 35% with respect to the Scherrer method was obtained, as shown in TiO_2_ NPs, where W–H crystallite sizes were either 3–5 nm bigger than the sizes of the Scherrer equation [45].

**Figure 9:**
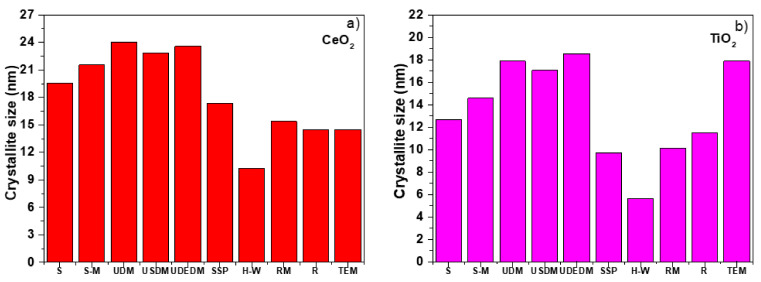
Comparison of the average crystallite size obtained by different estimation methods S (Scherrer method), RM (Rietveld Refinement), and R (μ-Raman) for CeO_2_ (**a**) and TiO_2_ NPs (**b**).

Finally, Figure 9a,b shows crystallite size values derived from the different methods based on the analysis of line profiles using PXRD, showing a strong variation in the three W–H models for nanoCeO_2_, as reflected in the data fit shown in the previous figures. In the case of the H–W method, it showed an underestimation in the crystallite size, while on the other hand, the RM was consistent with the size–strain method showing a slight variation of the integral breadth method. The RM and Raman methods were most accurate in the case of nanoCeO_2_, while W–H seemed to be the more accurate in the case of TiO_2_. However, W–H did not include the domain size distribution model and could not be used for the accurate determination of crystallite size. These results provided reliability in the RM and allowed its use to obtain average crystallite sizes, being one of the most important parts in the characterization of nanomaterials.

## 4. Conclusions

In this work, an extended analysis of XRD data for CeO_2_ and TiO_2_ NPs was presented, using crystallite size estimation methods. Among them were the Scherrer method, the Monshi method, the W–H model, UDM, UDEDM, SSP, and H–W methods. All of them suggested an important isotropic broadening, assuming Lorentzian and Gaussian profile contributions allowed to estimate the crystallite size and micro deformation of physical parameters. However, the method of Scherrer and W–H had less precision for the determination of the crystallite size for the presented metallic nano oxides by not considering the real TEM PSD. Furthermore, the crystallite size was calculated using the RM. For that, the IRF function was considered and obtained from the refinement of the standard Al_2_O_3_. By employing the RM, it was possible to carry out the refinement of CeO_2_ and TiO_2_ nanopowders, corroborating the phases of cubic nanoCeO_2_ and TiO_2_ anatase using the TCH profile and, hence, allowing the calculation of microstructural parameters using the SHP. This SHP allowed to obtain the domain size distribution of the crystallites. After comparing all the presented models, it was found for nanoCeO_2_ that the average crystallite sizes determined by the SHP and Raman methods were close to the results obtained by TEM, but for the TiO_2_, the W–H method was the closest model giving the smallest value of R^2^ and corroborating the anisotropic size model that suggested that each NP is made up of two or at least three crystallites. We presented a detailed XRD characterization that strongly correlated with the Raman and TEM analyses. This perspective can be used in future work in order to analyze and estimate accurately the crystallite size distributions present in NPs as prepared by different physical and chemical methods as well.

## Figures and Tables

**Table 1:** Microstructural parameters of the CeO_2_ and TiO_2_ NPs obtained by several crystalline size analysis methods.

		Samples	CeO_2_	TiO_2_
Scherrer method		*D* (nm)	19.6 (2)	12.7 (2)
		R^2^	0.99	0.26
Modified Scherrer method		*D* (nm)	21.6 (3)	14.6 (2)
		R^2^	0.99	0.27
Williamson–Hall method	UDM	*D* (nm)	24 (9)	17.9 (8)
		*ε* × 10^−3^	0.75 (1)	2.30 (2)
		R^2^	0.86	0.02
	USDM	*D* (nm)	22.8 (1)	17 (6)
		*ε* × 10^−3^	0.56 (1)	1.95(1)
		*σ* (TPa) × 10^−4^	1.51(5)	2.48(2)
		R^2^	0.56	0.08
	UDEDM	*D* (nm)	23.6 (1)	18.5(8)
		*ε*× 10^−7^	1.26 (1)	7.42(1)
		*σ* (TPa) × 10^−4^	1.85 (4)	3.1 (2)
		*U* (TJm^−3^) × 10^−7^	0.63 (3)	3.71(1)
		R^2^	0.75	0.08
Size–strain plot method		*D* (nm)	17.4 (5)	9.7 (2)
		*ε*× 10^−3^	0.77 (1)	4.83 (1)
		R^2^	0.999	0.81
Halder–Wagner method		*D* (nm)	10.3 (8)	5.6 (2)
		R^2^	0.999	0.992
		*ε* × 10^−3^	97.2 (7)	17.1 (5)
Rietveld Refinement (SHP)		*D* (nm)	15.4 (1)	10.1 (2)
		*ε*	32.9 (2)	110.7 (5)
μ-Raman		*D* (nm)	14.5 (1)	11.5 (1)
TEM		*D* (nm)	14.5 (5)	17.9 (5)

**Table 2:** Rietveld refinement parameters of CeO_2_ and TiO_2_ samples using the FullProf program: cell parameters, cell volume, and agreement factors. $R_{p}\;\left(\%\right)$ and $R_{wp}\;\left(\%\right)$ are the profile residual and the weighted profile residual factors, respectively, used to verify the Rietveld refinement quality. The goodness of fit, chi-square (*χ*^2^). The K coefficients correspond to the CeO_2_ phase and the *Y* coefficients to the anatase phase.

Refinement Parameters	nanoCeO_2_	nanoTiO_2_
Profile	TCH	TCH
$a\;\left(Å\right)$	5.4106	3.7839
$b\;\left(Å\right)$	5.4106	3.7839
$c\;\left(Å\right)$	5.4106	9.5017
$\alpha \;\left(Å\right)$	90	90
$\beta \;\left(Å\right)$	90	90
$\gamma \;\left(Å\right)$	90	90
$V\;\left({Å}^{3}\right)$	158.396	136.045
$K_{00},\;Y_{00}$	0.087 (2)	0.000 (2)
$K_{41},\;Y_{20}$	0.098 (3)	0.535 (2)
$K_{61},\;Y_{21^{+}}$	0.000 (2)	0.387 (5)
$K_{62},\;Y_{21^{-}}$	−1.119 (8)	−0.967 (2)
$K_{81},\;Y_{22^{+}}$	−0.091 (3)	−0.167 (3)
$Y_{22^{-}}$	-	0.473 (2)
FWHM parameters		
U	0.085	4.384
V	−0.41	−2.610
W	0.014	0.921
Global average size (nm)	15.4 (1)	10.1 (2)
$R_{p}\;\left(\%\right)$	6.08	5.46
$R_{wp}\;\left(\%\right)$	5.79	5.55
*χ^2^*	2.99	1.69

## Data Availability

The simulated data of the present research can be provided upon reasonable request from juan.ramos5@unmsm.edu.pe.

## Supplementary Materials

The following are available online at https://www.mdpi.com/article/10.3390/nano11092311/s1. Figure S1. Instrumental resolution function obtained from the standard corundum. Figure S2. The pure PXRD pattern of CeO_2_ (a) and TiO_2_ NPs (b). Figure S3. The modified W–H analysis of CeO_2_ (a) and TiO_2_ NPs (b), assuming USDM. The modified W–H analysis of CeO_2_ (c) and TiO_2_ NPs (d), assuming UDEDM.
