# Supplementary material for: Strain and Grain Size Determination of CeO2 and TiO2 Nanoparticles: Comparing Integral Breadth Methods versus Rietveld, μ-Raman, and TEM
Source: Nanomaterials (Basel). 2021 Sep 6;11(9):2311. doi: 10.3390/nano11092311 (PMC8469540; doi:10.3390/nano11092311)
Supplement: Supplementary file 1 [file nanomaterials-11-02311-s001.zip › nanomaterials-1350747-supplementary.pdf]

Supplementary Material

Strain and Grain Size Determination of CeO_2 and TiO_2 Nanoparticles: Comparing Integral Breadth Methods Versus Rietveld, μ-Raman, and TEM

Yamerson Canchanya-Huaman [1], Angie F. Mayta-Armas [1], Jemina Pomalaya-Velasco [1], Yéssica Bendezú-Roca [1], Jorge Andres Guerra [2] and Juan A. Ramos-Guivar [3,*]

1. Laboratorio de No Metálicos, Facultad de Ingeniería Química, Universidad Nacional del Centro del Perú (UNCP), Av. Mariscal Ramón Castilla No 3909, El Tambo, Huancayo 12000, Peru; yamerson2016@gmail.com (Y.C.-H.); armasfiorella23@gmail.com (A.F.M.-A.); jpomalayavelasco@gmail.com (J.P.-V.); ybendezu@uncp.edu.pe (Y.B.-R.)
2. Departamento de Ciencias, Sección Física, Pontificia Universidad Católica del Perú, Av. Universitaria 1801, Lima 15088, Peru; guerra.jorgea@pucp.edu.pe
3. Grupo de Investigación de Nanotecnología Aplicada para Biorremediación Ambiental, Energía, Biomedicina y Agricultura (NANOTECH), Facultad de Ciencias Físicas, Universidad Nacional Mayor de San Marcos, Av. Venezuela Cdra 34 S/N, Ciudad Universitaria, Lima 15081, Peru

* Correspondence: juan.ramos5@unmsm.edu.pe; Tel.: +51-1-914728212

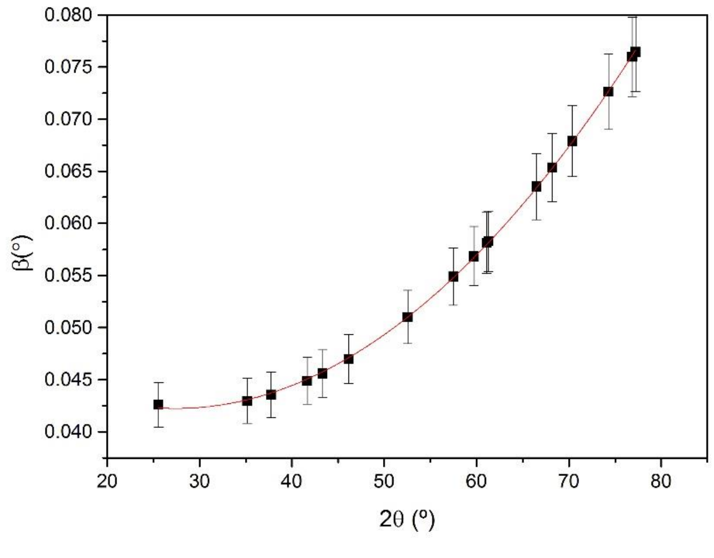

Figure S1. Instrumental resolution function obtained from standard corundum.

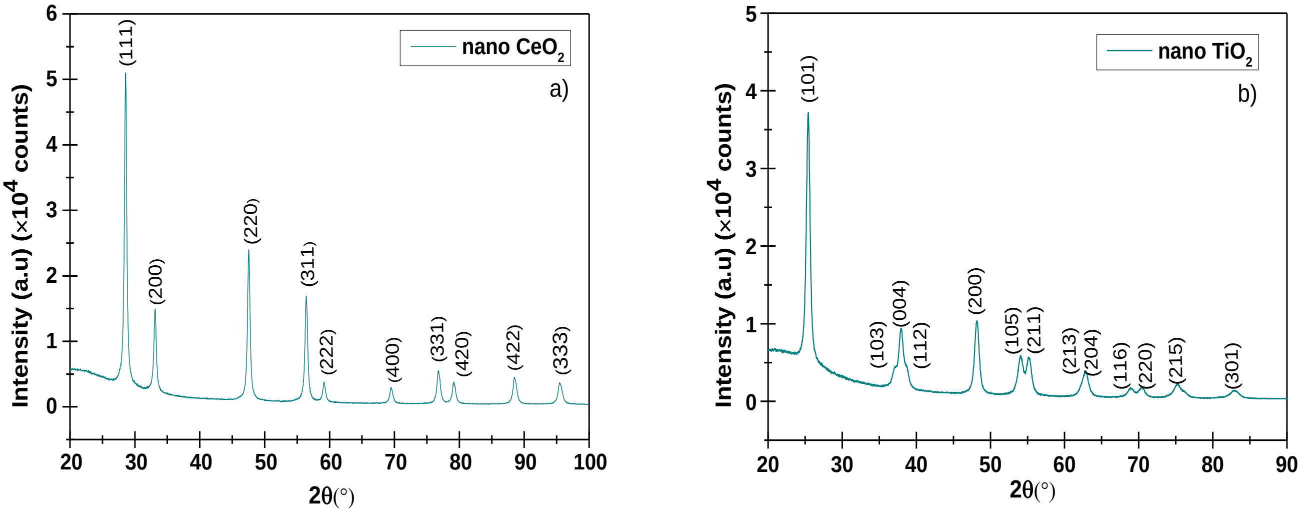

Figure S2. Pure p-XRD pattern of CeO_2 (**a**) and TiO_2 NPs (**b**).

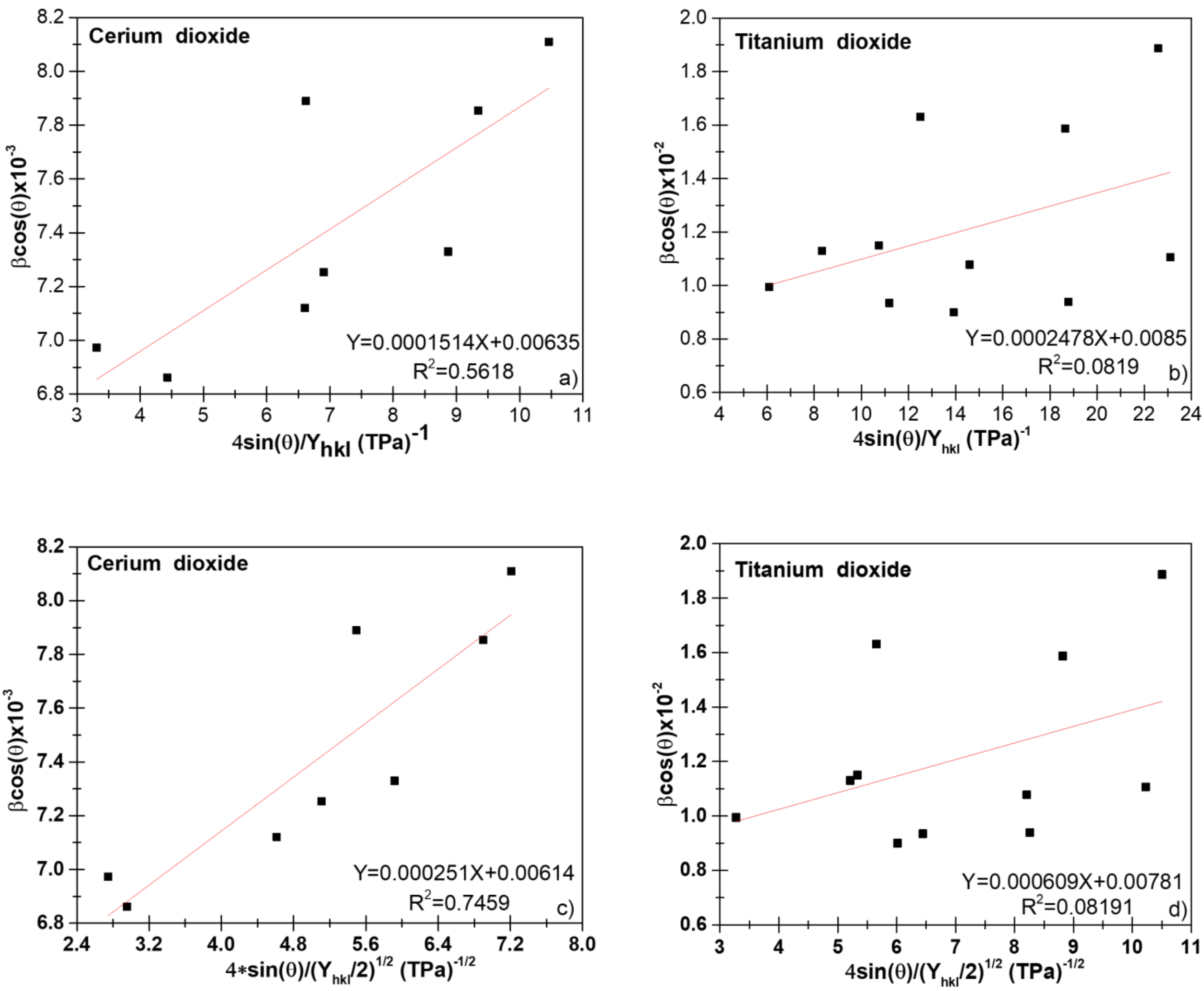

Figure S3. The modified W-H analysis of CeO_2 (**a**) and TiO_2 NPs (**b**), assuming USDM. The modified W-H analysis of CeO_2 (**c**) and TiO_2 NPs (**d**), assuming UDEDM.
